# An optimized block-based intuitionistic fuzzy framework for multi-focus image fusion

**DOI:** 10.3389/frai.2026.1905582

**Published:** 2026-08-28

**Authors:** Ragavendirane M.S, J. Reegan Jebadass, S. Dhanasekar, S. Lakshmanan

**Affiliations:** 1 Department of Mathematics, School of Advanced Sciences, Vellore Institute of Technology, Chennai, Tamil Nadu, India; 2 Department of Mathematics, Sathyabama Institute of Science and Technology, Chennai, Tamil Nadu, India

**Keywords:** entropy, image fusion, intuitionistic fuzzy generator, intuitionistic fuzzy image, multi-focus image fusion

## Abstract

**Introduction:**

Multi-focus image fusion aims to generate a single all-in-focus image by combining multiple images captured at different focal depths. However, accurately identifying focused and blurred regions remains challenging due to real-world focus transitions and the uncertainties inherent between sharp and defocused areas.

**Methods:**

To address these limitations, this article presents a new multi-focus image fusion method employing a novel intuitionistic fuzzy generator. In the proposed framework, an input image is initially converted into an intuitionistic fuzzy image (IFI), followed by a fusion rule termed the optimized block-based partitioning and defocusing synthesis algorithm to generate the final fused image. The utilization of IFIs facilitates the management of uncertainties associated with the membership and non-membership degrees of an image.

**Results:**

The proposed fusion algorithm demonstrates superior performance compared with existing state-of-the-art methods in terms of entropy, average gradient, and spatial frequency.

**Discussion:**

Analytical experiments and comparative evaluations demonstrate that the proposed approach achieves improved visual quality and effectively addresses the uncertainties associated with focused and defocused regions in multi-focus image fusion.

## 1 Introduction

Digital image processing plays a pivotal role in a diverse array of applications, including pattern recognition, clinical imaging, and remote sensing. Image fusion is an evolving paradigm within the field of image processing (Sridhar, 2011) that has transformed multi-source data integration by combining inputs from disparate sensors into a unified, highly informative representation. The resulting fused image quality exceeds that of any single source image, offering enhanced and more relevant information for subsequent quantitative and qualitative analysis. Recent technological advances have elevated image fusion to a foundational tool across spatial and frequency domains (Stathaki, 2011). As an example, (Mandhare et al. 2013) focused on improving the spatial and spectral information in remote sensing images by applying pixel-level fusion methods based on discrete wavelet transform techniques. Different fusion techniques, such as averaging, multiplication, Brovey transforms, and discrete wavelet transform (DWT), are assessed.

(Bahri and Shiwani 2016) presented a new fusion technique utilizing multi-resolution singular value decomposition (MSVD), demonstrating superior compositing performance over wavelet-based methods in terms of peak signal-to-noise ratio (PSNR), mean squared error (MSE) across multifocal images, computed tomography, multi-sensor satellite image, human brain magnetic resonance imaging datasets, etc. The MSVD algorithm can be further enhanced to improve the PSNR and MSE performance. Besides that, (Diwakar et al. 2021), the authors have discussed the comparative study between the stationary wavelet transform (SWT) and DWT frameworks using spatial frequency metrics for medical image fusion. From the study, generally, SWT outperforms DWT in terms of the high decomposition levels, resulting in better quality and reduced blurriness in the fused images. (Zhang et al. 2021) proposed a multi-focus image fusion method integrating superpixel clustering with a unified low-rank representation model, effectively mitigating spatial and boundary artifacts.

In recent years, deep learning techniques have significantly advanced the field of image fusion. (Zhang and Ma 2021) introduced a squeeze-and-decomposition network (SDNet) for real-time image fusion that demonstrates superior performance in terms of subjective visual quality and qualitative metrics. SDNet utilizes adaptive decision blocks, intensity- and gradient-level loss functions, and a squeeze fusion loss to enhance fusion performance through joint optimization. Meanwhile, (Ying et al., 2023) proposed a region-aware image fusion (RaIF) framework for RGB and near-infrared images that generates fused images with enhanced visual clarity in the sky and vegetation regions. In addition, RaIF can serve as a refinement module for deep learning-based frameworks and effectively recover salient highlighted regions. In (Tang et al. 2023), the authors presented a combination of a scene-illumination disentangled network and a texture-contrast enhancement fusion network to generate fused images with natural color and significant contrast, termed DIVFusion, which enhances fused image quality even under low-lighting conditions. Recently, (Fan and Wei 2024) proposed a hybrid generative adversarial network (GAN)-based multi-spectral image fusion model that enhances high-dimensional feature extraction and alignment by integrating gradient moment matching with adversarial learning—thereby addressing key limitations of traditional fusion methods. Similarly, a study in (Hou et al. 2025) introduced an improved deep learning-based fusion framework that combines advanced feature extraction and optimization strategies to enhance the quality and consistency of multi-source image fusion. Meanwhile, (Li et al. 2026) proposed a lightweight framework for infrared and low-light night vision image fusion, addressing the limitations of visible spectrum imagery in extremely dark environments. To synthesize recent approaches in image fusion, the authors (Singh et al., 2023; Zhou et al., 2023) conducted a comprehensive literature reviews of various image fusion techniques, detailing with their applications and listed their pros and cons.

On the other hand, uncertainties are an unavoidable factor when fusing multiple images, often leading to diminished visual quality (e.g., inconsistencies in pixel intensity levels). The concept of fuzzy sets, introduced by (Zadeh et al. 1996), incorporates each element associated with its membership function (Klir and Yuan, 1995), and it is also very helpful in associating additional information about the images to deal with the random state of pixels during image processing. In scholarly publications, numerous studies have explored using the concept of fuzzy sets and their extensions for image fusion (Chaira and Ray, 2017). For example, (Singh et al. 2004) discussed the various applications of fuzzy logic and neuro-fuzzy architectures for image fusion in different medical fields. The authors in (Zhu and Yang 2008) have proposed a fusion algorithm that merges fuzzy logic and wavelet transform for infrared and visible images. Inverse wavelet transform is performed to reconstruct the fused image, and weighted average coefficients are computed based on the relevance of the coefficients. In (Das and Kundu 2013), the authors approached medical image fusion using multi-scale geometric analysis, and a fuzzy-adaptive reduced pulse-coupled neural network, demonstrating better performance in terms of preserving image details and avoiding image degradation. The enhanced image fusion algorithm proposed in (Nandal and Rosales 2016) that utilizes directional contrast rules in the fuzzy transform domain to overcome the limitations of contrast enhancement, which often result in poor contrast quality in fused images.

Subsequently, (Atanassov 2012) introduced a concept known as intuitionistic fuzzy sets (IFS) which is an extension of Zadeh's fuzzy sets that incorporates both the memberships and non-membership functions to capture richer informational functions to capture richer informational nuances from images. Numerous researchers have leveraged this framework for image fusion methodology and put forth various methods. (Balasubramaniam and Ananthi 2014) presented a new method for image fusion using IFS, which is very useful in handling uncertainties in the image data. It further employs entropy to optimize the membership and non-membership degrees and reassemble the data into blocks according to the information in the blackness and whiteness of the image. In terms of brightness, contrast, and other performance parameters, the experimental findings reveal that their recommended technique outperforms other existing methods. Subsequently, (Palanisami et al. 2022) proposed an approach to fusing techniques for multimodal medical images using IFS that achieves higher visual clarity, enhanced contrast, and freedom from artifacts. Moreover (Jiang et al. 2023b) introduced a new similarity measure based on IFS for medical image fusion integrated with the Laplacian pyramid decomposition. This method decomposes images into high- and low-frequency sub-images, leveraging the proposed similarity measure to effectively extract the key image features. The authors in (Jiang et al. 2023a) have proposed a new entropy measure for medical image fusion using IFSs combined with a joint Gaussian curvature filter. This approach focuses on the potential and applications of the entropy measure of the IFS, calculating the imprecise features of medical images transformed by source image pixels. Recently, in (Jebadass and Balasubramaniam 2023) developed a block separation blur unification (SBU) technique for fusion based on interval type-2 IFSs. The outcome was examined using several other image fusion methods already in use. This technique can find applications across various real-world domains and fields.

The previously reviewed techniques that we discussed earlier are based on intuitionistic fuzzy measures and explain the usage of intuitionistic fuzzy generating functions (IFG) like (Bustince et al., 2000; Chaira, 2021) in image fusion. In addition, such IFGs widely applied to enhance low-light images such as Chinnappan and Sundaram (2024); (Ragavendirane and Dhanasekar 2025a); (Selvam et al. 2024); (Jebadass and Balasubramaniam 2024); (Ragavendirane and Dhanasekar 2025b) and so on, but in this study, we introduce a new intuitionistic fuzzy generating function derived from the previously mentioned functions of Sugeno's class. This newly introduced function can be employed to achieve IFIs with superior performance in contrast to other existing IFGs.

The literature review has examined a wide spectrum of image fusion techniques across both crisp and fuzzy perspectives, each presenting particular strategies for combining image data, and also explored a wide range of applications that are far-reaching and span a diverse range of fields. This article presents a novel intuitionistic fuzzy generator (NIFG)-based fusion approach for multifocus images that allows a flexible representation of the images as membership, non-membership, and hesitation components. These kinds of image representations can be very helpful in dealing with uncertainty and imprecision in image data, leading to better results in image analysis. The proposed method also provides an enhanced view of the IFIs by consolidating them into a single image to provide a more comprehensive and improved visual understanding.

The article's key contributions are:

- This research introduced a new optimized block-based fusion strategy fused with NIFG for multi-focus image fusion. The main objective of the NIFG is to obtain the intuitionistic fuzzy components of the source images to obtain IFI.
- Unlike existing fuzzy-based fusion methods (Ananthi and Balasubramaniam, 2015; Balasubramaniam and Ananthi, 2014; Jebadass and Balasubramaniam, 2023) that rely on IFSs, interval-valued IVIFS and type-2 IVIFS. In this method, images are fused using the optimized block partitioning and defocusing synthesis (OBPDS) algorithm. This algorithm consists of three main categories: optimized block partition, image defocusing, and block-image synthesis, followed by the defuzzification processes.
- With this OBPDS algorithm, the output images have greater dependability and higher quality because of their optimized block partitioning with maximum entropy, average gradient (AG), and spatial frequency (SF) levels.
- From the simulation results, the proposed approach provided better results than existing methods in (Ananthi and Balasubramaniam 2015), (Bavirisetti and Dhuli 2016), (Chai et al. 2017), (Diwakar et al. 2021), (Jebadass and Balasubramaniam 2023), (Kaur et al. 2021), and (Li et al. 2018). Specifically, the suggested approach minimizes the occurrence of fusion artifacts compared with alternative image fusion methods.

**Figure 1:**
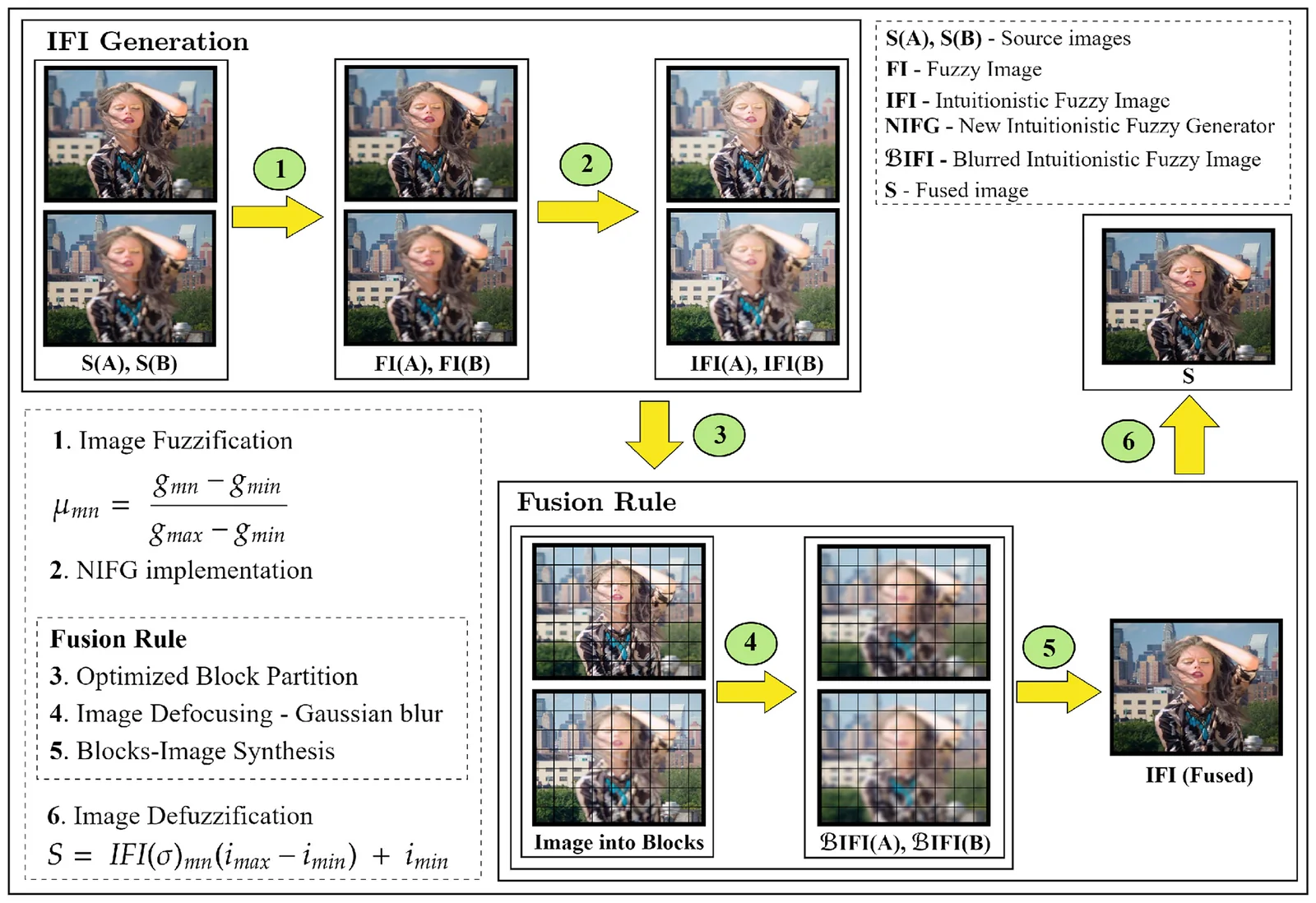
Workflow of OBPDS algorithm.

A limitation of the recommended fusion technique is that when the output image ignores some information, its absence occurs in similar locations for all the source images during their integration. Figure 1 shows a schematic representation of the workflow for the proposed model. The Comparative study of the proposed method with other fusion techniques such as principal component analysis (PCA), wavelet transform (WT), stationary wavelet transform (SWT), two-scale (TS), pixel average (PA), image fusion using IFS representation model (Ananthi's IFI), and block-SBU fusion using interval type-2 intuitionistic fuzzy representation (Reegan's IVT2IFI) in terms of entropy, AG, and SF showcases superior efficiency.

The OBPDS method can be utilized in various applications, such as in medical and clinical applications; this fusion technique can be instrumental in integrating multi-sensor images such as MRI, CT scan reports, etc. In remote sensing, multiple images of the same scene are captured and sent through a satellite from space to Earth for research purposes. Some of these images contain incomplete information regarding specific aspects of the scene. In such cases, this image fusion technique assists by fusing appropriate images and providing a better visual understanding of the scene.

In the following manuscript, Section 2 comprises preliminary details required for the proposed method. Section 3 describes the methodology of the proposed fusion technique. Section 4 evaluates the performance metrics of the proposed approach in comparison to existing image fusion algorithms. Finally, Section 5 concludes the article.

## 2 Preliminaries

### 2.1 Fuzzy set

Let *𝕊* be the universe of discourse and G be a subset of *𝕊*, a fuzzy set $\tilde{G}$ in *𝕊* is defined as $\tilde{G}=\{g,\mu_{\tilde{G}}\left(g\right)$ ∀*g*∈*𝕊*}, where $\mu_{\tilde{G}}$ is the membership function of g in G, and $\mu_{\tilde{G}}\left(g\right)\to \left[0,1\right]$ represents the degree of membership function.

### 2.2 Intuitionistic fuzzy set

Let *𝕊* be the universe of discourse and G be a subset of *𝕊*. An intuitionistic fuzzy set (IFS) ${\tilde{G}}_{I}$ in *𝕊* is defined as ${\tilde{G}}_{I}=\{g,\mu_{G}\left(g\right),\nu_{G}\left(g\right)$ ∀*g*∈*S*}, where μ_*G*_ is called as the membership function100000000 and ν_*G*_ is called as the non-membership function of g respectively. Both μ_*G*_(*g*) and ν_*G*_(*g*) ∈ [0,1] and 0 ≤ μ_*G*_(*g*)+ν_*G*_(*g*) ≤ 1.

Π_*G*_(*g*) is called the hesitation part or intuitionistic index and it is denoted by:


$$\begin{array}{l} \Pi_{G}\left(g\right)=1-\left(\mu_{G}\left(g\right)+\nu_{G}\left(g\right)\right), \end{array}$$ (1)

where, 0 ≤ Π_*G*_(*g*) ≤ 1. Similarly, the degree of membership and non-membership for an IFS can be represented as,


$$\begin{array}{l} \mu_{G}\left(g\right)=1-\nu_{G}\left(g\right)-\Pi_{G}\left(g\right), \end{array}$$ (2)

$$\begin{array}{l} \nu_{G}\left(g\right)=1-\mu_{G}\left(g\right)-\Pi_{G}\left(g\right), \end{array}$$ (3)

where, μ_*G*_(*g*)+ν_*G*_(*g*)+Π_*G*_(*g*) = 1.

### 2.3 Image blurring and defocus analysis

In image processing, blurring is characterized by a reduction in sharpness and spatial clarity in an image, leading to a more softened or diffused visual appearance. This process involves averaging or smoothing pixel values within an image, often over a neighborhood or a specific region, to remove high-frequency components (such as noise or fine structural features) and produce a more uniform or softened image (Yitzhaky and Kopeika, 1997).

In this article, Gaussian blur is utilized. It works by applying a two-dimensional Gaussian function, which creates a smooth, bell-shaped curve, to the pixels in the image. The result is a smoothing effect where each pixel's value is replaced by a weighted average of its neighboring pixels, with the weights determined by the Gaussian function.

Gaussian blur of an image can be determined by,


$$\begin{array}{l} G\left(x,y\right)=\frac{1}{2\pi \left(Sd\right)}e^{-\frac{x^{2}+y^{2}}{2\left({Sd}^{2}\right)}} \end{array}$$ (4)

Here, *G*(*x, y*) represents the value of the Gaussian function at the point (x, y), and *Sd* is the standard deviation of the distribution.

### 2.4 Image quality evaluation metrics

Image quality evaluation metrics are quantitative measures used to assess the performance of image processing algorithms. In the context of multi-focus image fusion, these metrics evaluate the effectiveness of the fused image in preserving important information from the source images such as structural details and overall visual clarity. In this work, the following metrics are utilized.

#### 2.4.1 Structural similarity index measure

The Structural Similarity Index Measure (SSIM) serves as a metric for assessing the similarity between two images. It is specifically designed to gauge the structural similarity, luminance, and contrast details between an original image and a modified version. SSIM is widely applied in image processing and computer vision, particularly for evaluating the effectiveness of techniques such as image and video compression, denoising, and image enhancement algorithms.


$$\begin{array}{l} SSIM\left(S\left(A\right),S\left(B\right)\right)=\frac{\left(2\zeta_{1}\zeta_{2}+x_{1}\right)\left(2\lambda_{12}+x_{2}\right)}{\left({\zeta_{1}}^{2}+{\zeta_{2}}^{2}+x_{1}\right)\left({\lambda_{1}}^{2}+{\lambda_{2}}^{2}+x_{2}\right)}, \end{array}$$ (5)

where *x*_1_ and *x*_2_ are non-negative constants. ζ_1_ and ζ_1_ denotes the average gray levels of images *S*(*A*) and *S*(*B*). λ_1_ and λ_2_ denotes the variance of *S*(*A*) and *S*(*B*). λ_12_ indicates the covariance of *S*(*A*) and *S*(*B*) respectively.

#### 2.4.2 Entropy of an image

Entropy is a key concept in image processing (Jebadass and Balasubramaniam, 2024) that quantifies the amount of information or uncertainty contained within a given image. It is a statistical metric that characterizes the distribution of pixel intensities. High-entropy images exhibit a wider range of pixel intensities and a more complex texture, whereas low-entropy images display a more uniform distribution of pixel intensities, resulting in a simpler or more regular texture.


$$\begin{array}{l} E=-\overset{i}{\underset{x=1}{\sum }}\left(p\left(x_{i}\right)\times log_{2}p\left(x_{i}\right)\right),0\leq i\leq 255, \end{array}$$ (6)

where *p*(*x*_*i*_) is the co-appearance of intensity values of the image. *x* indicates different intensity values of the image.

#### 2.4.3 Average gradient

The average gradient measures the overall change in pixel intensity or color across an image. It reflects the degree of sharpness, edge definition, or transitions of the required image. The average gradient of an image is defined as follows:


$$\begin{array}{l} AG=\frac{\left(\sum |S_{mn}|\right)}{\left(W_{i}\times H_{i}\right)}, \end{array}$$ (7)

where, *S*_*mn*_ is the gradient magnitude of an image with pixel entries *m, n*. *W*_*i*_ is the width of an image. *H*_*i*_ is the height of an image.

#### 2.4.4 Spatial frequency

In image processing, spatial frequency (SF) quantifies the rate of spatial variation in pixel intensity or color across an image. It is a fundamental metric used to characterize the patterns, textures, and details present in an image. Sharp transitions, such as edges or fine details, are represented by high spatial frequencies, whereas gradual transitions, such as wide gradients or homogeneous regions, are represented by low spatial frequencies. The SF of an image is defined as follows:


$$\begin{array}{l} SF=\sqrt{RF^{2}+CF^{2}}, \end{array}$$ (8)

where RF and CF denotes the row and column frequency of the pixels arranged in an image.


$$\begin{array}{l} RF=\sqrt{\frac{1}{M\left(N-1\right)}\overset{M}{\underset{i=1}{\sum }}\overset{N-1}{\underset{j=1}{\sum }}{\left[f\left(i,j+1\right)-f\left(i,j\right)\right]}^{2}} \end{array}$$ (9)

$$\begin{array}{l} CF=\sqrt{\frac{1}{\left(M-1\right)N}\overset{M-1}{\underset{i=1}{\sum }}\overset{N}{\underset{j=1}{\sum }}{\left[f\left(i+1,j\right)-f\left(i,j\right)\right]}^{2}} \end{array}$$ (10)

## 3 Methodology

The following method is applied to fuse images from the same frame of different focal depths into a single image with IFS.

Algorithm 1 Pseudo code for optimized block partition. 
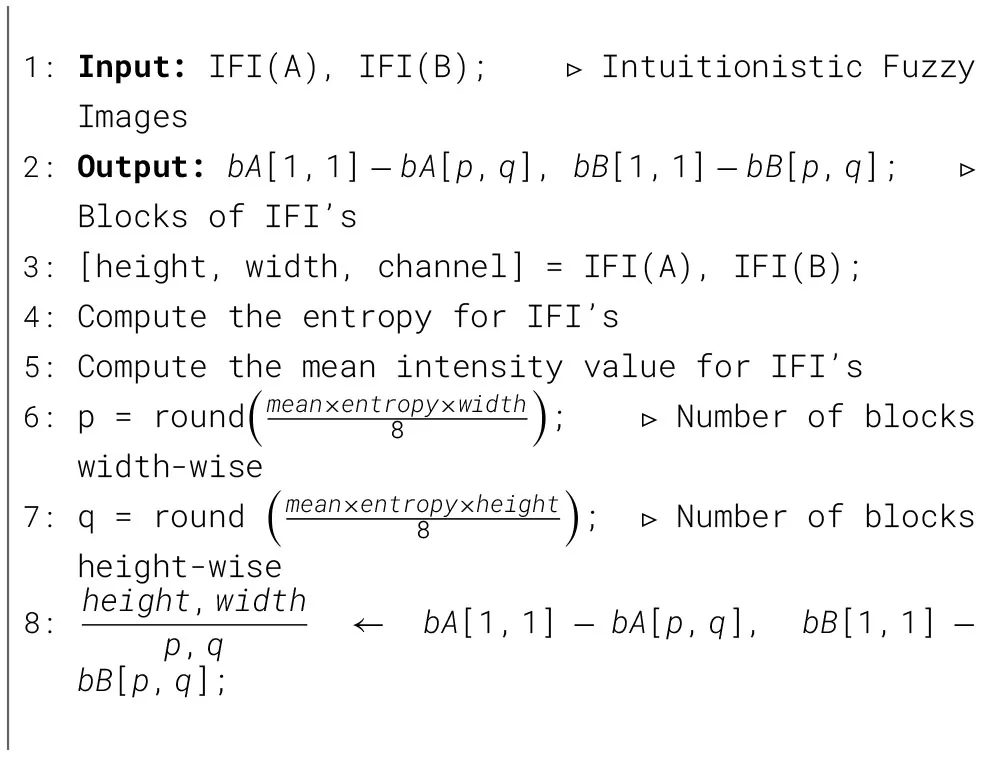

Algorithm 2 Pseudo code for the OBPDS. 
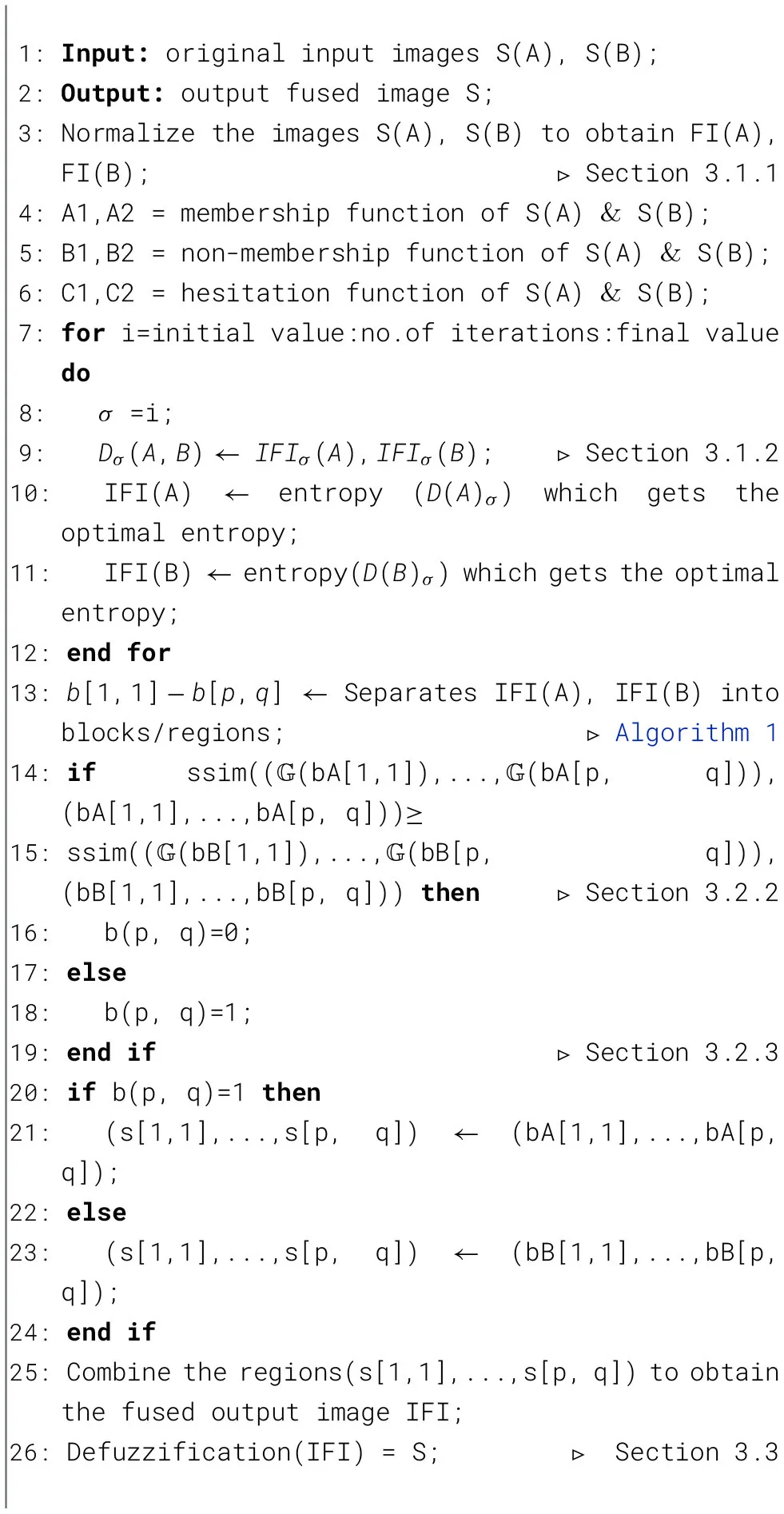

### 3.1 Construction of an intuitionistic fuzzy image

#### 3.1.1 Fuzzy image

At the outset, the input image *G* undergoes normalization to transform it into a fuzzy image (FI).


$$\begin{array}{l} \mu_{G_{mn}}=\frac{g_{mn}-g_{min}}{g_{max}-g_{min}}, \end{array}$$ (11)

where *g*_*mn*_ is the respective pixel level values, *g*_*max*_ and *g*_*min*_ denote the uppermost and lowermost pixel intensity values found in image *G*.

#### 3.1.2 Novel intuitionistic fuzzy generator

The transformation of an IFI from an FI is achieved by incorporating an IFG, which facilitates the representation of uncertainty and ambiguity inherent in the FI for a nuanced visual understanding. An IFG is function *n*(*g*):[0, 1] where *n*(*g*) ≤ 1−*g*, ∀, and 0 ≤ *g* ≤ 1. There are many IFGs, such as, Yager's (2009), Sugeno's (Murofushi et al., 2000), and Chaira's (2020), Chaira and Ray (2017), but for the interpretation of better results in the fusion's visual perception, an NIFG is utilized.

The characterization of the fuzzy complementary function (Chaira, 2021) is required to create a NIFG (Chaira, 2020). If *n* is a function *n*:[0, 1] → [0, 1], there exist a continuous function *f* such that *f*(0) = 0 and it is an increasing function,


$$\begin{array}{l} n\left(\mu_{G}\left(g\right)\right)=f^{-1}\left(f\left(1\right)-f\left(\mu \left(g\right)\right)\right). \end{array}$$ (12)

The fuzzy complement function (Higashi and Klir, 1982) can be written as,


$$\begin{array}{l} n\left(\mu_{G}\left(g\right)\right)=f^{-1}\left(f\left(0\right)-f\left(\mu \left(g\right)\right)\right). \end{array}$$ (13)

$$\begin{array}{l} f\left(\mu_{G}\left(g\right)\right)=\frac{1}{\sigma^{2}}log\left(1+\frac{\mu_{G}\left(g\right)}{16}{\left(4\sigma -3\right)}^{2}\right),\sigma >0. \end{array}$$ (14)

Moreover, considering μ_*G*_(*g*) as *g* for simplicity,


$$\begin{array}{l} f\left(g\right)=\frac{1}{\sigma^{2}}log\left(1+\frac{g}{16}{\left(4\sigma -3\right)}^{2}\right),\sigma >0. \end{array}$$ (15)

$$\begin{array}{ll} f\left(0\right) & =\frac{1}{\sigma^{2}}log\left(1\right)=0,\\ f\left(1\right) & =\frac{1}{\sigma^{2}}log\left(1+{\left(\sigma -\frac{3}{4}\right)}^{2}\right). \end{array}$$

Let the inverse function of *f*(*g*) can be written as,


$$\begin{array}{l} f^{-1}\left(g\right)=\frac{e^{\sigma^{2}g}-1}{{\left(\sigma -\frac{3}{4}\right)}^{2}}. \end{array}$$ (16)

From Equation 12, we can write,


$$\begin{array}{l} n\left(g\right)=f^{-1}\left(\frac{1}{\sigma^{2}}log\left(\frac{1+{\left(\sigma -\frac{3}{4}\right)}^{2}}{1+\frac{g}{16}{\left(4\sigma -3\right)}^{2}}\right)\right), \end{array}$$ (17)

substitute Equation 17 in Equation 16. The NIFG we get,


$$\begin{array}{l} n\left(g\right)=\frac{1-g}{1+\frac{g}{16}{\left(4\sigma -3\right)}^{2}},\sigma >0, \end{array}$$ (18)

The membership function for the IFI is as follows:


$$\begin{array}{ll} \mu_{mn}^{\prime } & =1-\frac{1-\mu_{mn}}{1+\frac{\mu_{mn}}{16}{\left(4\sigma -3\right)}^{2}}, \end{array}$$

$$\begin{array}{l} =\frac{\mu_{mn}\left(1+{\left(\sigma -\frac{3}{4}\right)}^{2}\right)}{1+\frac{\mu_{mn}}{16}{\left(4\sigma -3\right)}^{2}}. \end{array}$$ (19)

The non-membership function for the IFI is as follows:


$$\begin{array}{ll} \nu_{mn}^{\prime } & =\frac{1-\mu_{mn}^{\prime }}{1+\frac{\mu_{mn}^{\prime }}{16}{\left(4\sigma -3\right)}^{2}},\\ =\frac{1-\left(\frac{\mu_{mn}\left(1+{\left(\sigma -\frac{3}{4}\right)}^{2}\right)}{1+\frac{\mu_{mn}}{16}{\left(4\sigma -3\right)}^{2}}.\right)}{1+{\left(\sigma -\frac{3}{4}\right)}^{2}\left(\frac{\mu_{mn}\left(1+{\left(\sigma -\frac{3}{4}\right)}^{2}\right)}{1+\frac{\mu_{mn}}{16}{\left(4\sigma -3\right)}^{2}}.\right)},\\ =\frac{1-\mu_{mn}}{1+\mu_{mn}\left(\sigma^{4}-3\sigma^{3}+\frac{35}{8}\sigma^{2}-\frac{75}{16}\sigma +\frac{369}{256}\right)}, \end{array}$$

$$\begin{array}{ll} \nu_{mn}^{\prime } & =\frac{1-\mu_{mn}}{1+\frac{\mu_{mn}}{8}{\left(4\sigma -3\right)}^{2}+\frac{\mu_{mn}}{16}{\left(4\sigma -3\right)}^{4}}. \end{array}$$ (20)

Therefore, from Equation 1, the hesitation part of the image with dimension (*m, n*) can be written in the form of


$$\begin{array}{l} \Pi_{mn}=1-\mu_{mn}^{\prime }-\nu_{mn}^{\prime }. \end{array}$$ (21)

#### 3.1.3 Intuitionistic fuzzy image

Once hesitation (Equation 21), member (Equation 19), and non-member (Equation 20) aspects of an image have been identified. The IFI can be determined by


$$\begin{array}{l} IFI{\left(\sigma \right)}_{mn}=\mu_{mn}^{\prime }+\Pi_{mn}. \end{array}$$ (22)

In each input image, a set of candidate intuitionistic fuzzy images is generated by varying the parameter σ over the range [0, 1], i.e., {σ_1_, σ_2_, …, σ_*n*_}, where *n*∈ℕ and 0 ≤ σ_*n*_ ≤ 1, as shown in Figure 1. In the present work, 100 uniformly distributed σ values are considered, thus generating 100 candidate IFIs for each input image.

Next, the entropy of each candidate IFI is computed as described in Section 2.4.2, yielding the set {*E*_1_, *E*_2_, …, *E*_*n*_}, where 0 ≤ *E*_*n*_ ≤ 8. The upper bound of 8 corresponds to the maximum Shannon entropy of an 8-bit image with 256 intensity levels, i.e., log_2_(256) = 8. The optimization criterion is entropy, since it measures the amount of information and the intensity variation that is preserved in the generated IFIs. A candidate with a higher entropy preserves greater structural detail and useful focus-related information for the subsequent fusion stage.

Thus, the optimal parameter is determined by


$$\sigma^{*}=\arg\underset{\sigma_{n}}{\max}E_{n}.$$

**Figure 2:**
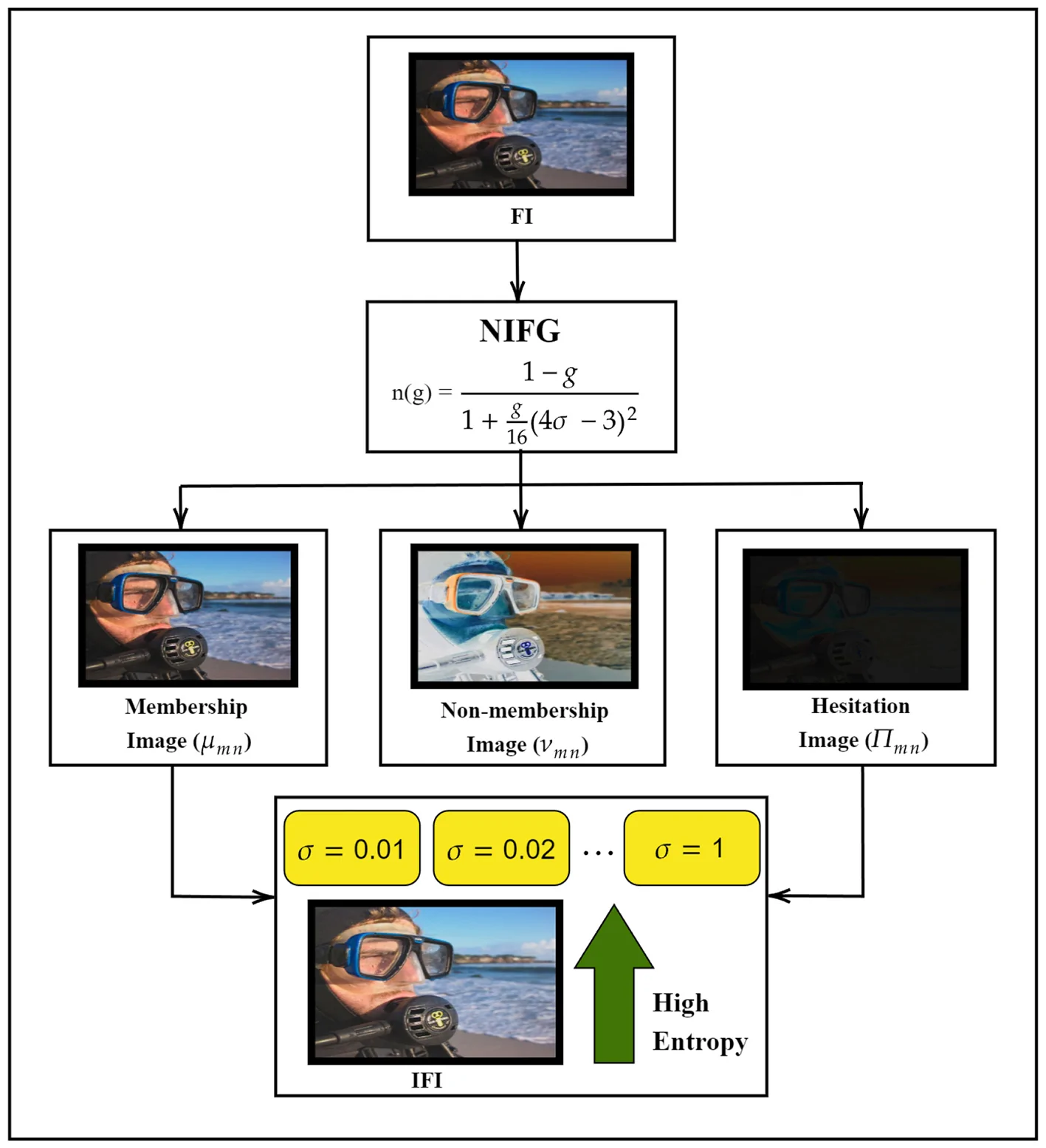
IFI formulation.

The proposed method can explore a variety of possible fuzzy representations by using multiple σ values instead of a single predefined parameter. The selection procedure reduces the possibility of picking a sub-optimal representation and enhances the robustness of the following fusion process. Figure 2 shows the visual formulation of the IFI obtained.

### 3.2 Fusion rule

#### 3.2.1 Optimized block partition

The resulting intuitionistic fuzzy images, IFI(A) and IFI(B), are adaptively partitioned into block matrices, which are denoted by *bA*[1, 1], …, *bA*[*p, q*] and *bB*[1, 1], …, *bB*[*p, q*], respectively. Unlike fixed-size partitioning, the proposed approach determines the number of blocks and their respective sizes based on the intrinsic characteristics of each input image.

Initially, the image size, normalized mean intensity, and Shannon entropy are calculated. The mean intensity reflects the general brightness distribution, while entropy measures the information content and structural variation of the image. These measures are combined to select optimal values for the horizontal and vertical block counts, *p* and *q*, respectively as described in Algorithm 1. The partitioning is thus adapted to the information content of the input image and allows the preservation of focused details during fusion.

The factor 8 in the equations for the block partitioning serves as a normalization constant for the entropy term. For an 8-bit image with 256 intensity levels, the maximum Shannon entropy is log_2_(256) = 8 bits. Therefore, the calculated entropy is divided by 8 to be in the range [0, 1]. This normalized entropy value, along with the normalized mean intensity, prevents any single term from dominating the partitioning process, enabling a balanced and data-driven estimation of *p* and *q*. Hence, the proposed optimal block-partitioning scheme produces image-dependent blocks rather than relying on a fixed block size.


$$\begin{array}{lll} IFI\left(A\right) & = & \left[\begin{array}{cccc} bA\left[1,1\right] & bA\left[1,2\right] & \cdots & bA\left[1,q\right]\\ bA\left[2,1\right] & bA\left[2,2\right] & \cdots & bA\left[2,q\right]\\ \vdots & \vdots & \cdots & \vdots \\ bA\left[p,1\right] & bA\left[p,2\right] & \cdots & bA\left[p,q\right] \end{array}\right]\\ IFI\left(B\right) & = & \left[\begin{array}{cccc} bB\left[1,1\right] & bB\left[1,2\right] & \cdots & bB\left[1,q\right]\\ bB\left[2,1\right] & bB\left[2,2\right] & \cdots & bB\left[2,q\right]\\ \vdots & \vdots & \cdots & \vdots \\ bB\left[p,1\right] & bB\left[p,2\right] & \cdots & bB\left[p,q\right] \end{array}\right] \end{array}$$

#### 3.2.2 Image de-focusing

The separated regions {*bA*[1, 1], ⋯ , *bA*[*p, q*]} and {*bB*[1, 1], ⋯ , *bB*[*p, q*]} of the IFI(A) and IFI(B) images are blurred (see, Section 2.3) and modified into {*G*(*bA*[1, 1]), ⋯ , *G*(*bA*[*p, q*])} and {*G*(*bB*[1, 1]), ⋯ , *G*(*bB*[*p, q*])} corresponding regions. All the blocks with blurred regions are combined and represented together as the *G*IFI(A) and *G*IFI(B) matrices, shown respectively. This process of removing focus throughout an image by blurring is termed image defocusing.


$$\begin{array}{lll} GIFI\left(A\right) & = & \left[\begin{array}{cccc} G\left(bA\left[1,1\right]\right) & G\left(bA\left[1,2\right]\right) & \cdots & G\left(bA\left[1,n\right]\right)\\ G\left(bA\left[2,1\right]\right) & G\left(bA\left[2,2\right]\right) & \cdots & G\left(bA\left[2,n\right]\right)\\ \vdots & \vdots & \cdots & \vdots \\ G\left(bA\left[p,1\right]\right) & G\left(bA\left[p,2\right]\right) & \cdots & G\left(bA\left[p,q\right]\right) \end{array}\right]\\ GIFI\left(B\right) & = & \left[\begin{array}{cccc} G\left(bB\left[1,1\right]\right) & G\left(bB\left[1,2\right]\right) & \cdots & G\left(bB\left[1,q\right]\right)\\ G\left(bB\left[2,1\right]\right) & G\left(bB\left[2,2\right]\right) & \cdots & G\left(bB\left[2,q\right]\right)\\ \vdots & \vdots & \cdots & \vdots \\ G\left(bB\left[p,1\right]\right) & G\left(bB\left[p,2\right]\right) & \cdots & G\left(bB\left[p,q\right]\right) \end{array}\right] \end{array}$$

The structural similarity index measure (SSIM) in Equation 5 is used to quantify the structural change caused by defocusing within each corresponding block of *IFI*(*A*) and *IFI*(*B*). The SSIM is calculated for each block between the corresponding block of the original intuitionistic fuzzy image and its Gaussian-blurred counterpart, producing the sets {α_1, 1_, α_1, 2_, …, α_*p, q*_} and {β_1, 1_, β_1, 2_, …, β_*p, q*_} of *IFI*(*A*) and *IFI*(*B*), respectively.

SSIM is selected because it compares local luminance, contrast, and structural information, making it well-suitable for analyzing focus-related changes. An out-of-focus or blurred block experiences a smaller structural change after further Gaussian blurring, yielding a relatively higher SSIM value. In contrast, a focused block contains richer edges and fine details, so additional blurring causes greater structural degradations, resulting in a lower SSIM value. The SSIM values α_*i, j*_ and β_*i, j*_ are then used to determine the blur characteristics of the corresponding blocks. This comparison enables the selection of complementary focused blocks from *IFI*(*A*) and *IFI*(*B*) to construct the final fused image.

#### 3.2.3 Blocks-image synthesis

After estimating the SSIM values for each respective block in the *IFI*s corresponding to their counterparts *GIFI*s, the most suitable block *s*[*p, q*] is chosen based on its highest SSIM value. This selection ensures that the chosen region retains the optimal perceptual quality and focal detail for the respective block.


$$\begin{array}{l} s\left[p,q\right]\;\Rightarrow \;max\left(\alpha_{p,q},\beta_{p,q}\right) \end{array}$$ (23)

Finally, all blocks are integrated back together to construct a final fused image *IFI* that can be obtained from this blocks-image synthesis process by positioning the appropriate regions on their respective module locations. This process of blocks-image synthesis is achieved by placing each selected block to its appropriate spatial location which preserves the modular structure and focus of the entire fused image. Matrix representation of the fused *IFI* is demonstrated below:

$$IFI=\left[\begin{array}{cccc} s\left[1,1\right] & s\left[1,2\right] & \cdots & s\left[1,q\right]\\ s\left[2,1\right] & s\left[2,1\right] & \cdots & s\left[2,q\right]\\ \vdots & \vdots & \cdots & \vdots \\ s\left[p,1\right] & s\left[p,2\right] & \cdots & s\left[p,q\right] \end{array}\right]$$

### 3.3 De-fuzzification

In image processing, defuzzification is the process of converting the fuzzy pixel intensity values into a crisp image representation. In the proposed approach, the pixel values are replaced in the source images in terms of fuzzy memberships to understand the compatibility and similarity between pixels in different images. Consequently, defuzzifying the fused image *IFI* into its crisp counterpart enables attainment of the best possible output image *S*. For further pieces of information, kindly refer to (Yagar 1980).

From Equation 11,


$$\begin{array}{l} S_{mn}=IFI_{mn}\left(g_{max}-g_{min}\right)+g_{min} \end{array}$$ (24)

### 3.4 The proposed OBPDS fusion method

The proposed OBPDS-based image fusion algorithm begins by considering two source images, *S*(*A*) and *S*(*B*), of dimensions *M*×*N*. Initially, both images are normalized and transformed into their corresponding fuzzy representations, *FI*(*A*) and *FI*(*B*). Subsequently, intuitionistic fuzzy images, *IFI*(*A*) and *IFI*(*B*), are generated by varying the parameter σ within the interval [0, 1]. For each possible value of σ, entropy is computed, and the intuitionistic fuzzy images corresponding to the optimal entropy values are selected. The obtained images are then partitioned into non-overlapping blocks using a block partition matrix. To identify focused regions, each block is subjected to an image defocusing operation, resulting in blurred versions of the intuitionistic fuzzy images. The Structural Similarity Index Measure (SSIM) is then computed between the original and blurred blocks of each image. Based on the maximum SSIM criterion, the most informative blocks are selected and combined through an image synthesis process to generate the fused intuitionistic fuzzy image. Finally, the fused intuitionistic fuzzy image is defuzzified to obtain the final fused output image, *S*. The complete workflow of the proposed method is illustrated in Figure 1, while its implementation details are presented in Algorithm 2.

## 4 Performance metrics and result implications

The proposed algorithm in this study was executed using MATLAB R2024a on a system equipped with an AMD Ryzen 5 7520U processor featuring Radeon graphics clocked at 2.80 GHz. The system was configured with 16GB of RAM, and a 512GB SSD for storage and operated on the Microsoft Windows 11 Home operating system.

**Data Description**

**Figure 3:**
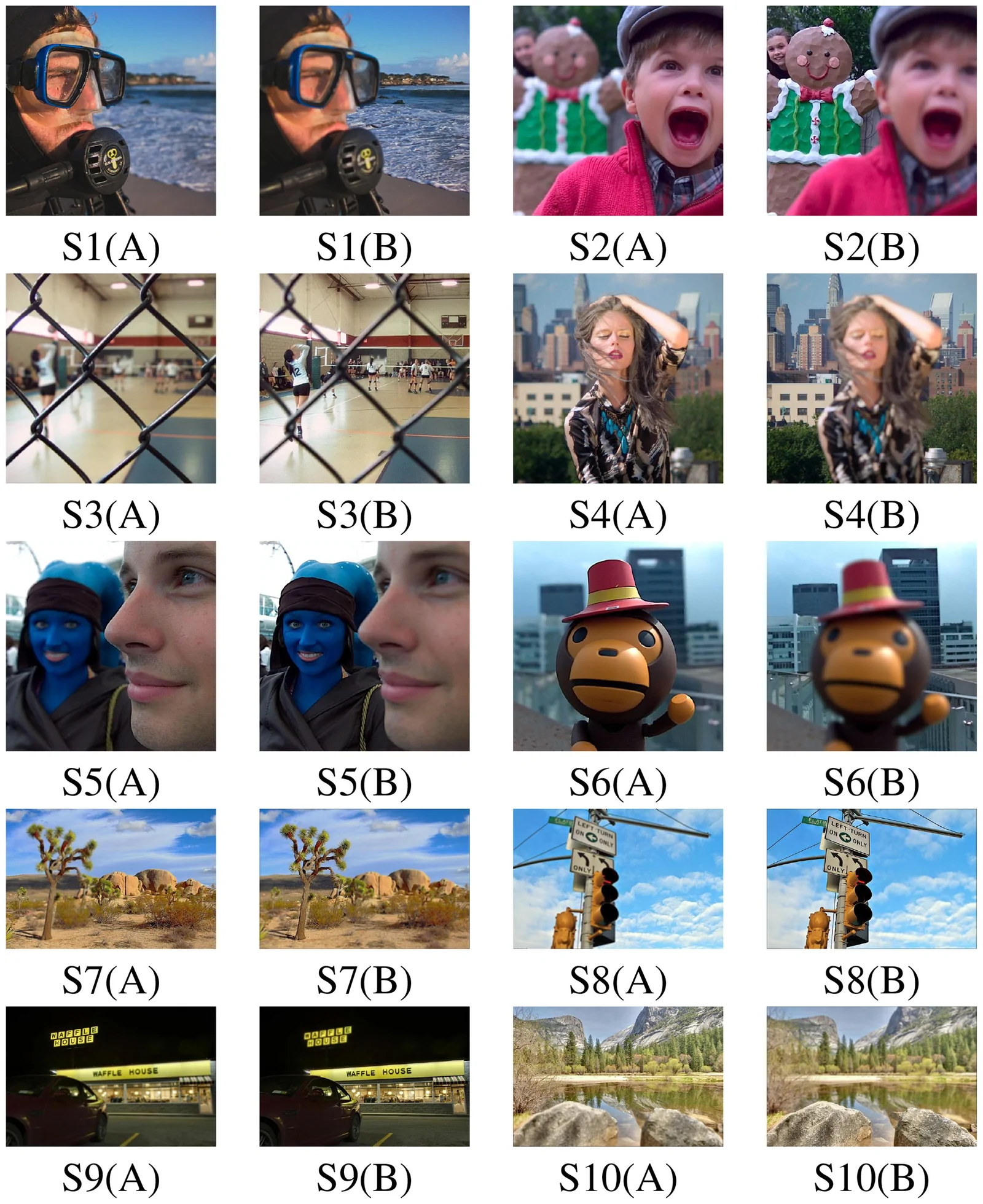
Source images from multi-focus image fusion data set.

- **Dataset:** Multi-Focus Image Fusion Dataset (Zafar et al., 2020).
- **Dataset size:** Nearly 500 color test images, with image sizes of 500 × 500 and 400 × 600 pixels, in .jpg and .png formats.
- **Image selection:** From 200 images considered for this study, 10 representative multi-focus image pairs were selected for processing and performance evaluation.
- **Selected pairs:** {(*S*_1_(*A*), *S*_1_(*B*)), (*S*_2_(*A*), *S*_2_(*B*)), …, (*S*_10_(*A*), *S*_10_(*B*))}, as shown in Figure 3.
- **Evaluation protocol:** The same image pairs and experimental settings were used for the proposed and comparison methods to ensure a fair evaluation.

The estimated results of the recommended algorithm were compared with seven different methods now in use, such as PCA, WT, SWT, TS, PA, Ananthi's IFI and Reegan's IVT2IFI (see Ananthi and Balasubramaniam, 2015; Bavirisetti and Dhuli, 2016; Chai et al., 2017; Diwakar et al., 2021; Jebadass and Balasubramaniam, 2023; Kaur et al., 2021; Li et al., 2018) via specific measures to evaluate their better-performing scale. Among these fusion algorithm, the article (Jebadass and Balasubramaniam, 2023) represents an interval type-2 intuitionistic fuzzy approach for image fusion. Due to the novelty in introducing the NIFG and an efficient fusion rule, the images are fused better in the intuitionistic fuzzy environment itself by the proposed methodology (Section 3.1.2) in all visual aspects.

For each processed image, a detailed visual comparison elaborately demonstrated with all other fusion techniques from Figures 4–13. All input images all are in RGB color space, and the quality of images was also evaluated in the same color space in the image spectrum through different metrics.

**Figure 4:**
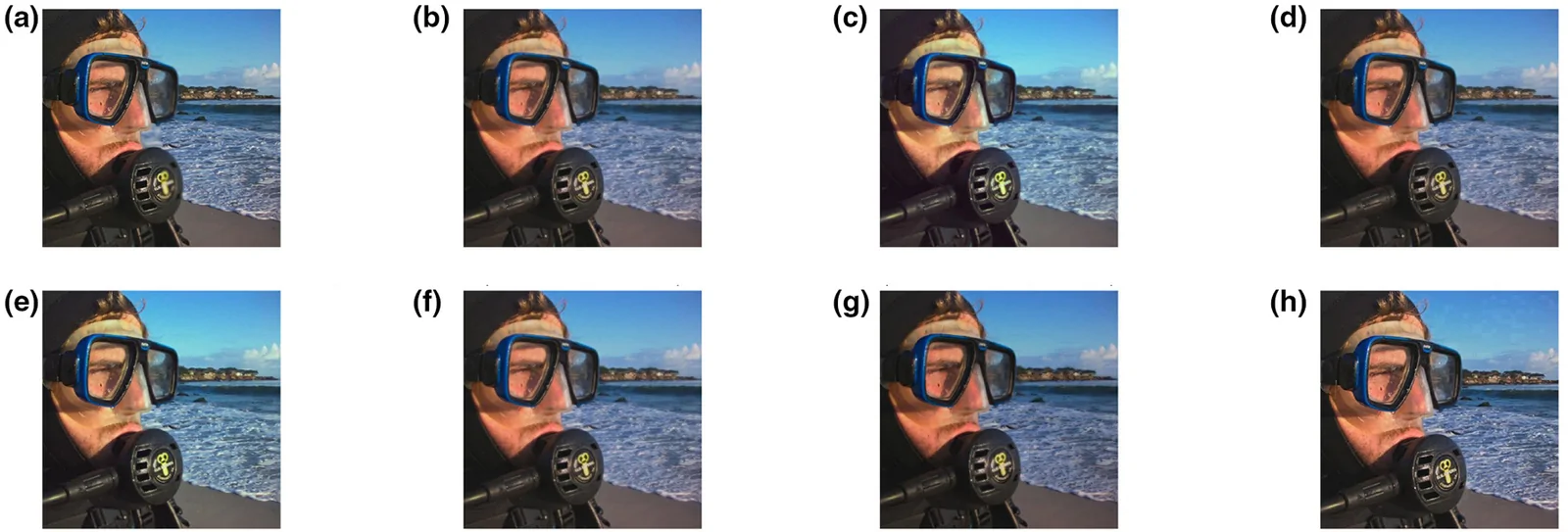
Output image S1 using OBPDS, PCA, DCHWT, WT, SWT, TS, PA, MSVD, and Reegan's IVT2IFI fusion methods. **(a)** (Jebadass and Balasubramaniam 2023). **(b)** (Li et al. 2018). **(c)** (Chai et al. 2017). **(d)** (Diwakar et al. 2021). **(e)** (Bavirisetti and Dhuli 2016). **(f)** (Kaur et al. 2021). **(g)** (Balasubramaniam and Ananthi 2014). **(h)** Proposed OBPDS.

In Figure 4, the fused outputs are shown for all 10 source images using the proposed OBPDS for image fusion. This method's performance is graphically evaluated with some other existing fusion techniques; hence, the superiority of the proposed OBPDS method is illustrated in Figures 14a–c, emphasizing the visual aspects of the images through E, AG, and SF. The graphs demonstrate that the proposed method is visually rich and qualitatively superior to other existing algorithms.

**Figure 5:**
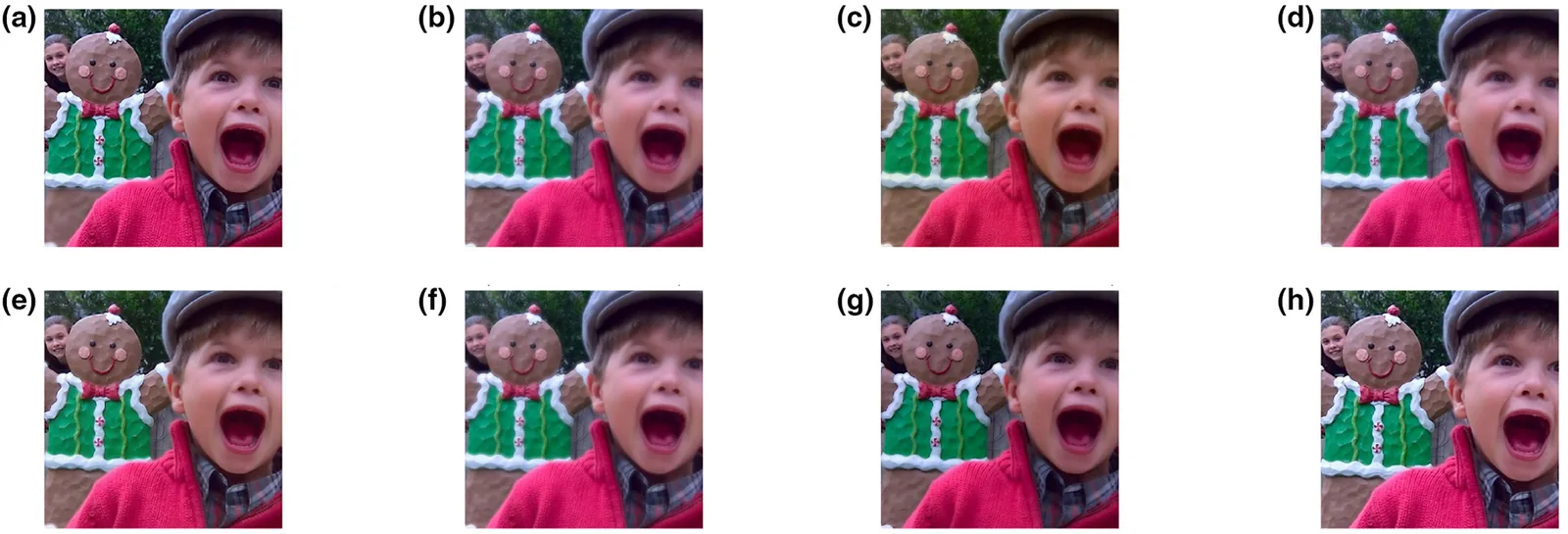
Output image S2 using OBPDS, PCA, DCHWT, WT, SWT, TS, PA, MSVD, and Reegan's IVT2IFI fusion methods. textbf(a) (Jebadass and Balasubramaniam 2023). **(b)** (Li et al. 2018). **(c)** (Chai et al. 2017). **(d)** (Diwakar et al. 2021). **(e)** (Bavirisetti and Dhuli 2016). **(f)** (Kaur et al. 2021). **(g)** (Balasubramaniam and Ananthi 2014). **(h)** Proposed OBPDS.

To quantitatively demonstrate the effectiveness and efficiency of the proposed methodology, detailed metric values are presented in Tables 1–3. These tables provide a comprehensive comparison across different evaluation criteria, highlighting the performance of the proposed approach relative to existing methods. Furthermore, a statistical analysis of the metrics with the dataset is demonstrated through the central tendency and dispersion measures using mean, median, standard deviation (SD), variance, and range via Tables 4–6.

**Table 1:** Entropy values of the output fused images obtained using the proposed fusion method and the comparative state-of-the-art fusion methods.

Source Images	(Li et al. 2018)	(Chai et al. 2017)	(Diwakar et al. 2021)	Bavirisetti and Dhuli, 2016	(Kaur et al. 2021)	Balasubramaniam and Ananthi, 2014	Jebadass and Balasubramaniam, 2023	Proposed OBPDS
S1	7.5547	7.5758	7.3250	7.5621	7.5498	7.5004	7.6147	**7.6898**
S2	7.7179	7.7302	7.6209	7.6995	7.7096	7.5265	7.7450	**7.8219**
S3	7.4898	7.5113	7.4423	7.5694	7.4805	7.6002	7.6084	**7.6473**
S4	7.6432	7.6529	7.5446	7.6460	7.6318	7.3356	7.6636	**7.7918**
S5	7.4831	7.4903	7.4295	7.4938	7.4844	7.3993	7.5371	**7.5677**
S6	7.6241	7.6349	7.5544	7.6284	7.6231	7.4093	7.6399	**7.6501**
S7	7.7977	7.8079	7.8286	7.8256	7.8023	7.6464	7.8257	**7.8613**
S8	7.4848	7.4958	7.4323	7.5327	7.4782	7.4070	7.5146	**7.5180**
S9	7.0359	7.0857	7.1719	7.2760	7.0392	6.8758	7.1798	**7.2191**
S10	7.1809	7.2469	7.1295	7.2978	7.1597	6.9575	7.3732	**7.3905**

Bold values indicate the effectiveness of the proposed fusion technique. Red, green, and blue values indicate the first, second, and third highest metric values, respectively.

**Table 2:** AG values of the output fused images obtained using the proposed fusion method and the comparative state-of-the-art fusion methods.

Source Images	(Li et al. 2018)	(Chai et al. 2017)	(Diwakar et al. 2021)	Bavirisetti and Dhuli, 2016	(Kaur et al. 2021)	Balasubramaniam and Ananthi, 2014	Jebadass and Balasubramaniam, 2023	Proposed OBPDS
S1	73.3635	80.6412	60.1489	81.0133	71.6870	65.9991	92.7463	**101.5440**
S2	42.8326	48.5406	38.4012	51.9664	41.2593	38.3576	62.5866	**59.8672**
S3	52.0168	59.3952	49.1948	71.6689	50.2578	51.2691	84.9216	**87.6078**
S4	35.0646	37.4466	31.5413	36.5269	34.4435	28.0595	40.3660	**38.1311**
S5	25.3317	27.9252	24.4950	31.6613	25.5278	26.4551	37.2471	**38.0714**
S6	29.9413	32.1205	28.4502	30.8439	29.9327	26.8413	34.7748	**34.9201**
S7	34.5524	38.8802	36.8333	41.6504	36.1803	30.8520	44.2837	**48.1352**
S8	57.0410	62.4666	53.0089	60.1553	55.4119	54.0084	69.5061	**70.5683**
S9	43.3081	51.0016	47.7257	56.4746	43.8108	41.0271	53.6072	**62.0239**
S10	69.3261	81.3359	63.5251	81.5555	66.0540	61.8701	102.4548	**105.5256**

Bold values indicate the effectiveness of the proposed fusion technique. Red, green, and blue values indicate the first, second, and third highest metric values, respectively.

**Table 3:** SF values of the output fused images obtained using the proposed fusion method and the comparative state-of-the-art fusion methods.

Source Images	(Li et al. 2018)	(Chai et al. 2017)	(Diwakar et al. 2021)	Bavirisetti and Dhuli, 2016	(Kaur et al. 2021)	Balasubramaniam and Ananthi, 2014	Jebadass and Balasubramaniam, 2023	Proposed OBPDS
S1	0.7628	0.7260	0.7276	0.6982	0.6649	0.7437	0.7366	**0.7957**
S2	0.3481	0.3272	0.3114	0.3556	0.3142	0.1248	0.3893	**0.4284**
S3	0.3191	0.3015	0.3602	0.3905	0.4082	0.3111	0.2777	**0.6883**
S4	0.6719	0.6784	0.6428	0.7120	0.6940	0.7100	0.6654	**0.7358**
S5	0.6660	0.6660	0.6620	0.6539	0.6555	0.6450	0.5892	**0.7028**
S6	0.7358	0.7412	0.7020	0.7359	0.6964	0.6817	0.7359	**0.7687**
S7	0.6660	0.6599	0.6555	0.6457	0.6757	0.5733	0.6737	**0.6800**
S8	0.5852	0.6106	0.5175	0.5518	0.5743	0.2974	0.7097	**0.7072**
S9	0.5618	0.5916	0.2294	0.4683	0.6080	0.6061	0.0000	**0.7134**
S10	0.6683	0.6743	0.6282	0.6616	0.6662	0.7395	0.4352	**0.7159**

Bold values indicate the effectiveness of the proposed fusion technique. Red, green, and blue values indicate the first, second, and third highest metric values, respectively.

**Table 4:** Central tendency and dispersion measures of entropy for the output fused images, including the mean, median, mode, standard deviation, variance and range, demonstrating the distribution and consistency of entropy values across the evaluated fusion methods.

Source Images	(Li et al. 2018)	(Chai et al. 2017)	(Diwakar et al. 2021)	Bavirisetti and Dhuli, 2016	(Kaur et al. 2021)	Balasubramaniam and Ananthi, 2014	Jebadass and Balasubramaniam, 2023	Proposed OBPDS
Mean	7.3041	7.3190	7.3000	7.3647	7.3013	7.1451	7.3809	**7.3832**
Median	7.4385	7.4295	7.4372	7.4882	7.4414	7.2445	7.5146	**7.5180**
SD	0.4575	0.4551	0.3923	0.4077	0.4567	0.5004	0.4256	**0.4382**
Variance	0.2093	0.2072	0.1539	0.1662	0.2086	0.2504	0.1812	**0.1920**
Range	2.1649	2.1374	2.1342	1.7336	1.8308	1.9898	2.7010	**2.1031**

Bold values indicate the results obtained using the proposed methodology.

**Table 5:** Central tendency and dispersion measures of SF for the output fused images, including the mean, median, mode, standard deviation, variance, and range, demonstrating the distribution and consistency of entropy values across the evaluated fusion methods.

Source Images	(Li et al. 2018)	(Chai et al. 2017)	(Diwakar et al. 2021)	Bavirisetti and Dhuli, 2016	(Kaur et al. 2021)	Balasubramaniam and Ananthi, 2014	Jebadass and Balasubramaniam, 2023	Proposed OBPDS
Mean	0.5502	0.5467	0.5368	0.5363	0.5452	0.5134	0.5232	**0.5568**
Median	0.5701	0.5788	0.5587	0.5683	0.5820	0.5367	0.5184	**0.5673**
SD	0.1441	0.1585	0.1343	0.1434	0.1465	0.1743	0.1888	**0.1580**
Variance	0.0208	0.0251	0.0180	0.0206	0.0215	0.0304	0.0356	**0.0250**
Range	0.6383	0.6259	0.6562	0.4982	0.7175	0.7947	0.6948	**0.7957**

Bold values indicate the results obtained using the proposed methodology.

**Table 6:** Central tendency and dispersion measures of AG for the output fused images, including the mean, median, mode, standard deviation, variance, and range, demonstrating the distribution and consistency of entropy values across the evaluated fusion methods.

Source Images	(Li et al. 2018)	(Chai et al. 2017)	(Diwakar et al. 2021)	Bavirisetti and Dhuli, 2016	(Kaur et al. 2021)	Balasubramaniam and Ananthi, 2014	Jebadass and Balasubramaniam, 2023	Proposed OBPDS
Mean	36.8473	36.5981	40.9598	35.8946	43.8051	49.9753	34.5705	**52.0640**
Median	34.3481	37.5087	33.2408	38.7871	34.1363	30.4042	43.2556	**46.1557**
SD	14.8207	16.7363	12.8715	16.6997	14.3698	14.2980	19.9392	**21.8763**
Variance	219.6537	280.1032	165.6745	278.8800	206.4905	204.4327	397.5700	**478.5726**
Range	62.3028	60.6249	68.8288	52.3217	70.2865	84.3325	60.3564	**93.1758**

Bold values indicate the results obtained using the proposed methodology.

Table 1 and Figure 14a represent the entropy of all 10 processed images, along with their comparable methods. According to the definition (Section 2.4.2), a maximum entropy value provides greater information in image fusion and is considered to be a good image. From this table, most of the images retaining the maximum entropy value, such as S1–S7, and S10, have the highest entropy value among all. S8 and S9 have the second highest entropy. Statistical properties of entropy with respect to central tendency and dispersion measures are calculated across all the comparison metrics and shown in Table 4.

Table 2 and Figure 14b present the AG values, which quantify information about the steepness of intensity variations in the resultant images (Section 2.4.3). It affirms that the proposed method has the maximum values, which are optimal and yield an average of 52.0640 for the entire dataset (Zafar et al., 2020). S1, S3, S5–S10 yield the maximum AG values than other methods, while S2 and S4 have the second-highest AG value. Quantitatively, Table 5 demonstrates the mean, median, SD, variance, and range of AG for the entire dataset through all other methodologies, and Figure 14f represents it graphically.

Figure 15 provides a qualitative comparison between the proposed method and a number of existing image fusion techniques. For detailed visual inspection, zoomed-in views of select regions and their corresponding Canny edge maps were displayed. From the zoomed regions, it is evident that the proposed method preserves finer texture details, sharper structural information, and better contrast compared with the competing methods. In addition, the edge maps exhibit more continuous and well-defined edges, indicating better edge preservation and structural fidelity. These visual results confirm the effectiveness and robustness of the proposed fusion approach in generating high-quality fused images.

**Figure 6:**
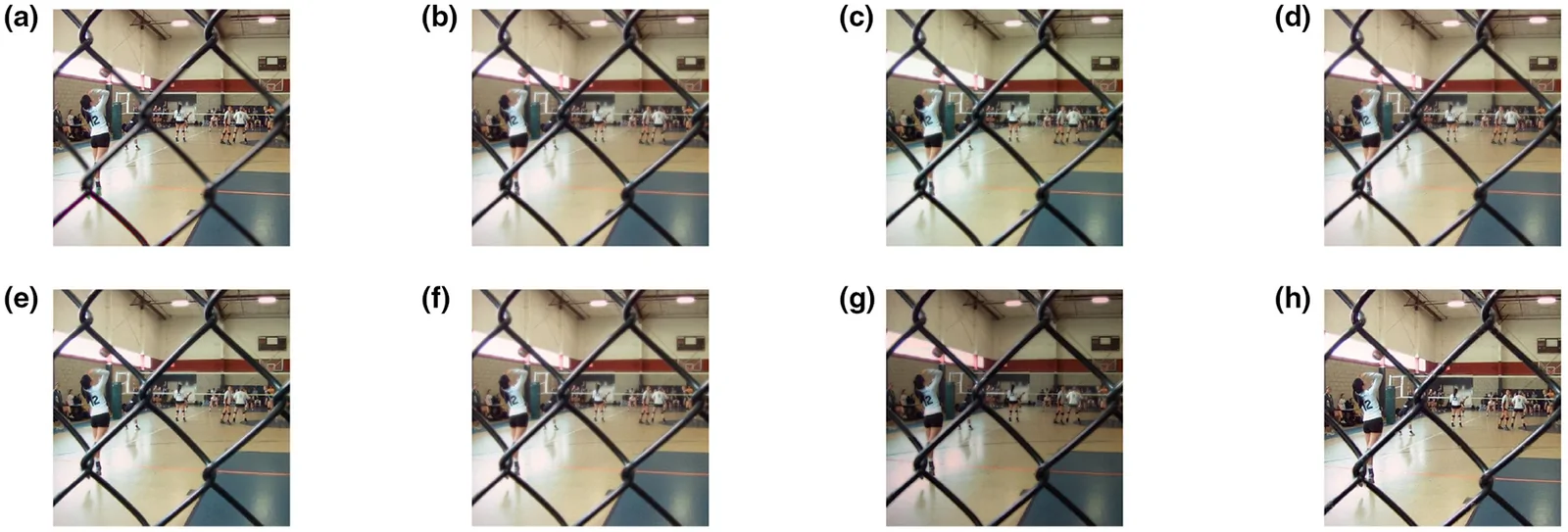
Output image S3 using OBPDS, PCA, DCHWT, WT, SWT, TS, PA, MSVD, and Reegan's IVT2IFI fusion methods. **(a)** (Jebadass and Balasubramaniam 2023). **(b)** (Li et al. 2018). **(c)** (Chai et al. 2017). **(d)** (Diwakar et al. 2021). **(e)** (Bavirisetti and Dhuli 2016). **(f)** (Kaur et al. 2021). **(g)** (Balasubramaniam and Ananthi 2014). **(h)** Proposed OBPDS.

**Figure 7:**
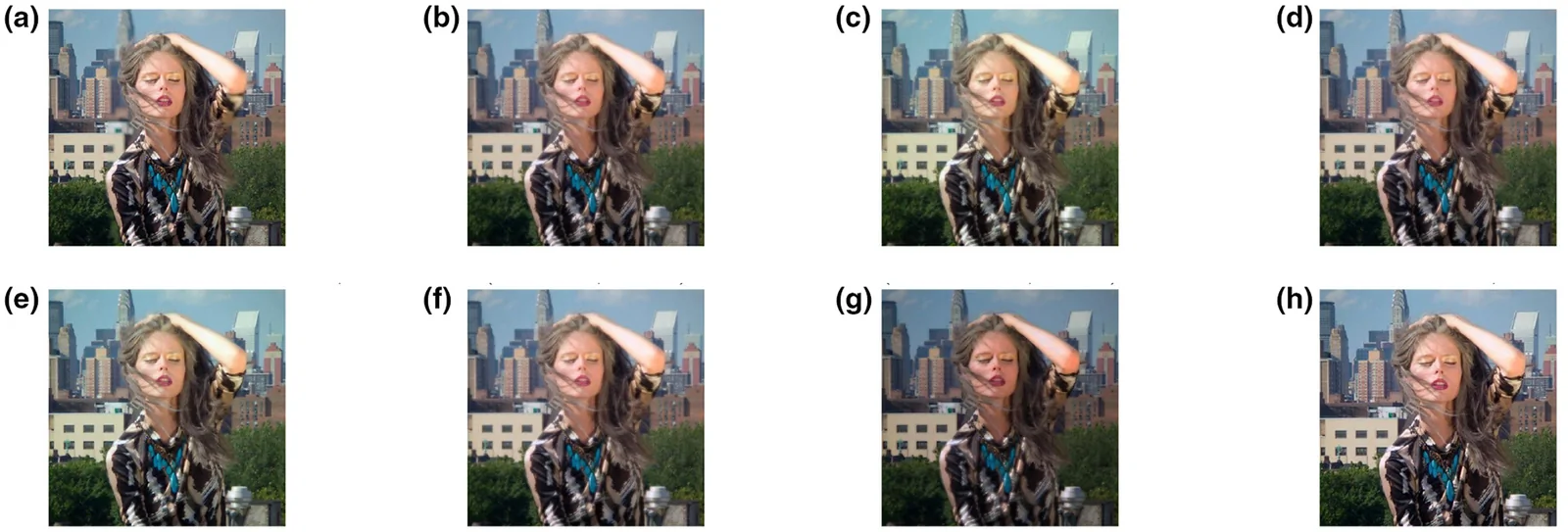
Output image S4 using OBPDS, PCA, DCHWT, WT, SWT, TS, PA, MSVD, and Reegan's IVT2IFI fusion methods. **(a)** (Jebadass and Balasubramaniam 2023). **(b)** (Li et al. 2018). **(c)** (Chai et al. 2017). **(d)** (Diwakar et al. 2021). **(e)** (Bavirisetti and Dhuli 2016). **(f)** (Kaur et al. 2021). **(g)** (Balasubramaniam and Ananthi 2014). **(h)** Proposed OBPDS.

**Figure 8:**
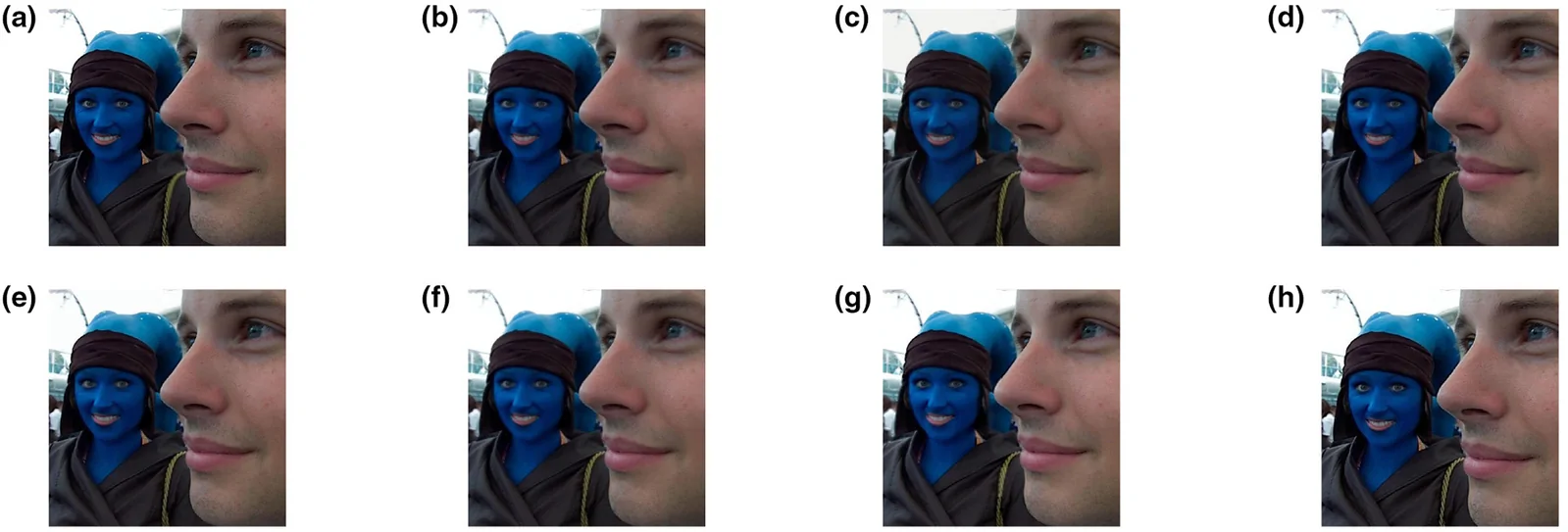
Output image S15using OBPDS, PCA, DCHWT, WT, SWT, TS, PA, MSVD, and Reegan's IVT2IFI fusion methods. **(a)** (Jebadass and Balasubramaniam 2023). **(b)** (Li et al. 2018). **(c)** (Chai et al. 2017). **(d)** (Diwakar et al. 2021). **(e)** (Bavirisetti and Dhuli 2016). **(f)** (Kaur et al. 2021). **(g)** (Balasubramaniam and Ananthi 2014). **(h)** Proposed OBPDS.

**Figure 9:**
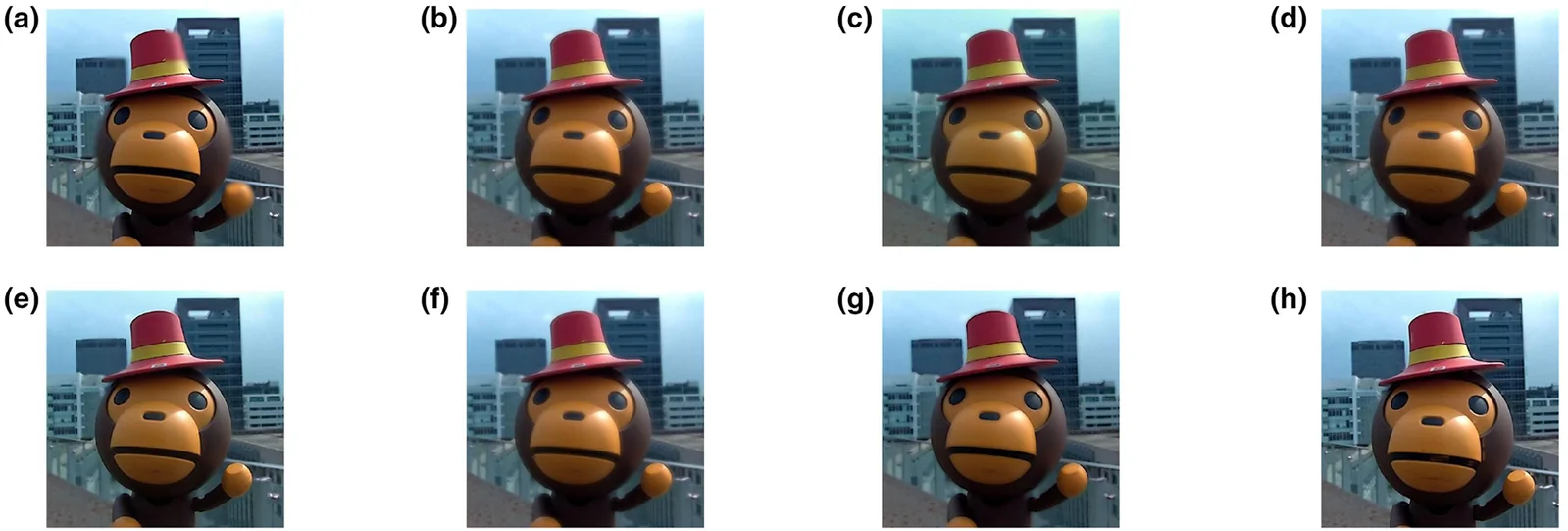
Output image S6 using OBPDS, PCA, DCHWT, WT, SWT, TS, PA, MSVD, and Reegan's IVT2IFI fusion methods. **(a)** (Jebadass and Balasubramaniam 2023). **(b)** (Li et al. 2018). **(c)** (Chai et al. 2017). **(d)** (Diwakar et al. 2021). **(e)** (Bavirisetti and Dhuli 2016). **(f)** (Kaur et al. 2021). **(g)** (Balasubramaniam and Ananthi 2014). **(h)** Proposed OBPDS.

**Figure 10:**
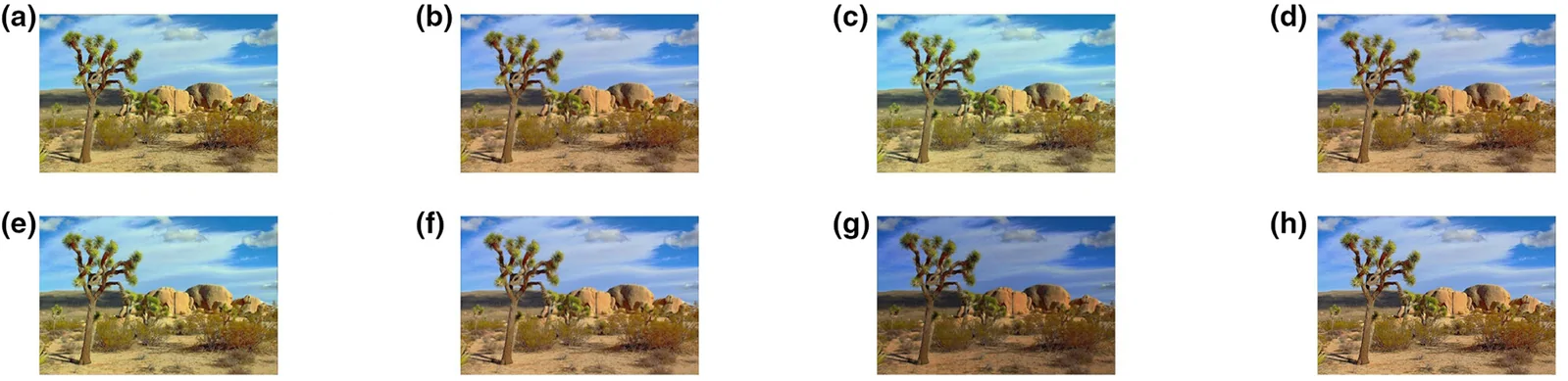
Output image S7 using OBPDS, PCA, DCHWT, WT, SWT, TS, PA, MSVD, and Reegan's IVT2IFI fusion methods. **(a)** (Jebadass and Balasubramaniam 2023). **(b)** (Li et al. 2018). **(c)** (Chai et al. 2017). **(d)** (Diwakar et al. 2021). **(e)** (Bavirisetti and Dhuli 2016). **(f)** (Kaur et al. 2021). **(g)** (Balasubramaniam and Ananthi 2014). **(h)** Proposed OBPDS.

**Figure 11:**
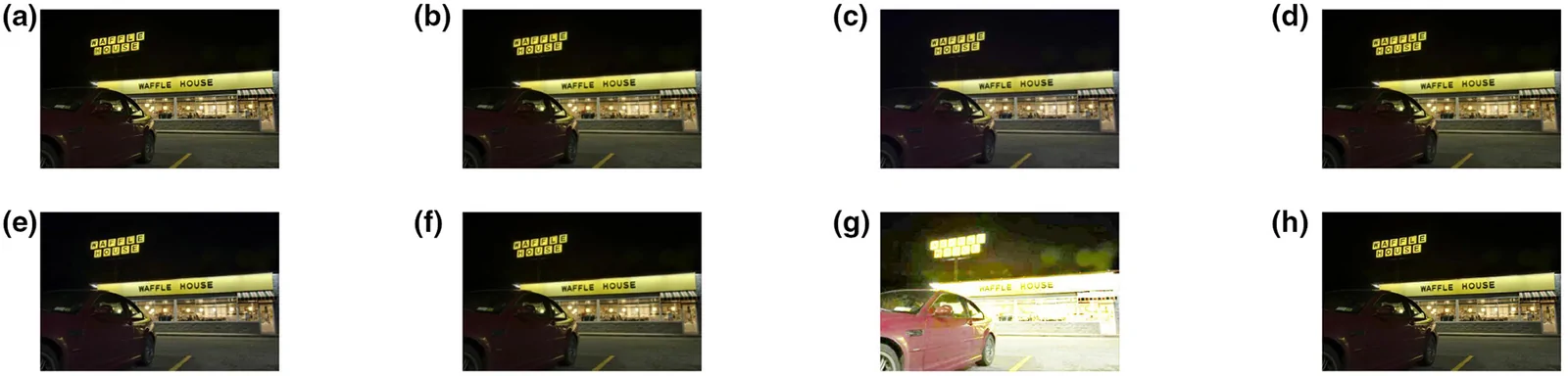
Output image S8 using OBPDS, PCA, DCHWT, WT, SWT, TS, PA, MSVD, and Reegan's IVT2IFI fusion methods. **(a)** (Jebadass and Balasubramaniam 2023). **(b)** (Li et al. 2018). **(c)** (Chai et al. 2017). **(d)** (Diwakar et al. 2021). **(e)** (Bavirisetti and Dhuli 2016). **(f)** (Kaur et al. 2021). **(g)** (Balasubramaniam and Ananthi 2014). **(h)** Proposed OBPDS.

**Figure 12:**
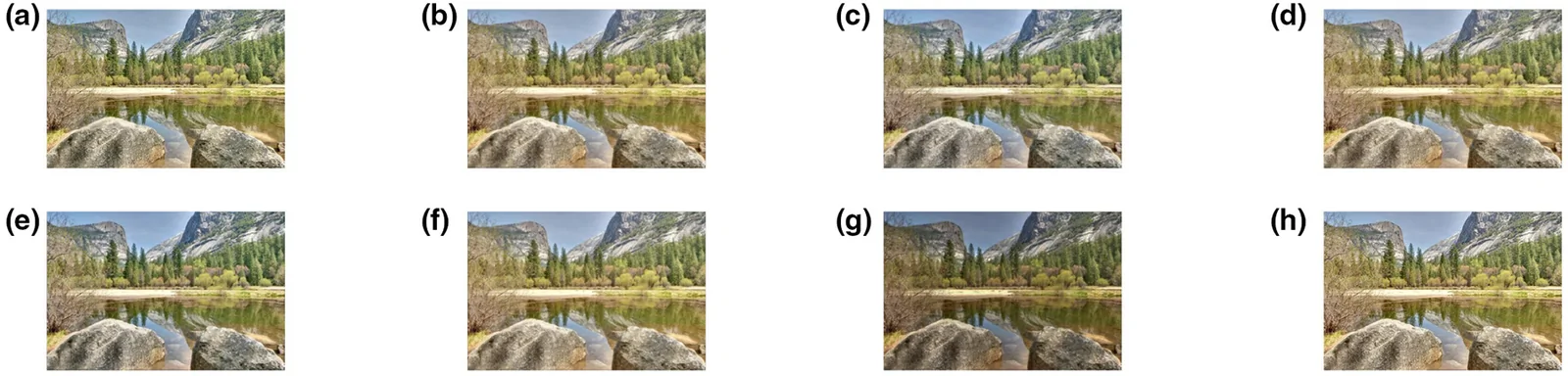
Output image S9 using OBPDS, PCA, DCHWT, WT, SWT, TS, PA, MSVD, and Reegan's IVT2IFI fusion methods. **(a)** (Jebadass and Balasubramaniam 2023). **(b)** (Li et al. 2018). **(c)** (Chai et al. 2017). **(d)** (Diwakar et al. 2021). **(e)** (Bavirisetti and Dhuli 2016). **(f)** (Kaur et al. 2021). **(g)** (Balasubramaniam and Ananthi 2014). **(h)** Proposed OBPDS.

**Figure 13:**
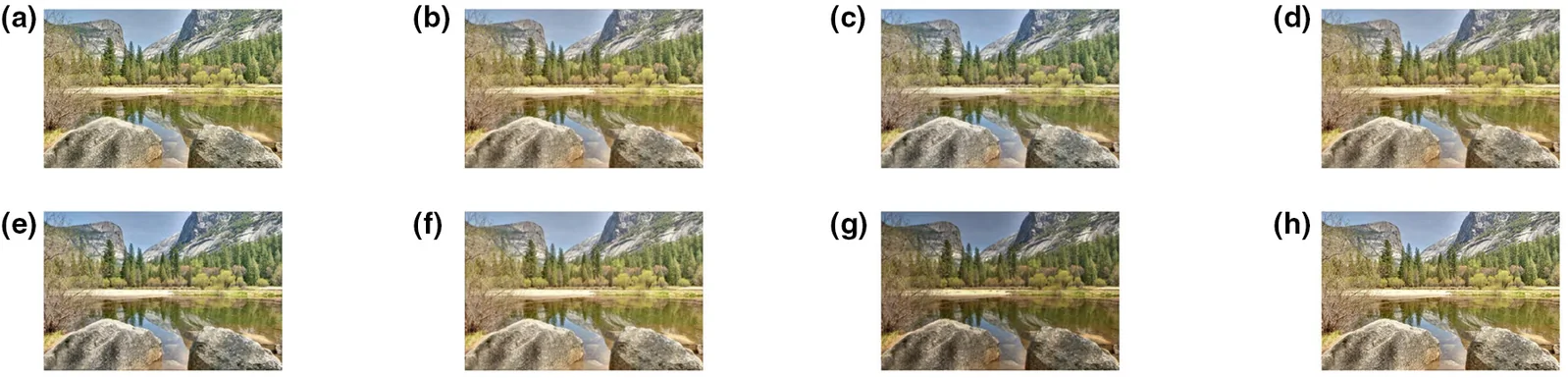
Output image S10 using OBPDS, PCA, DCHWT, WT, SWT, TS, PA, MSVD, and Reegan's IVT2IFI fusion methods. **(a)** (Jebadass and Balasubramaniam 2023). **(b)** (Li et al. 2018). **(c)** (Chai et al. 2017). **(d)** (Diwakar et al. 2021). **(e)** (Bavirisetti and Dhuli 2016). **(f)** (Kaur et al. 2021). **(g)** (Balasubramaniam and Ananthi 2014). **(h)** Proposed OBPDS.

**Figure 14:**
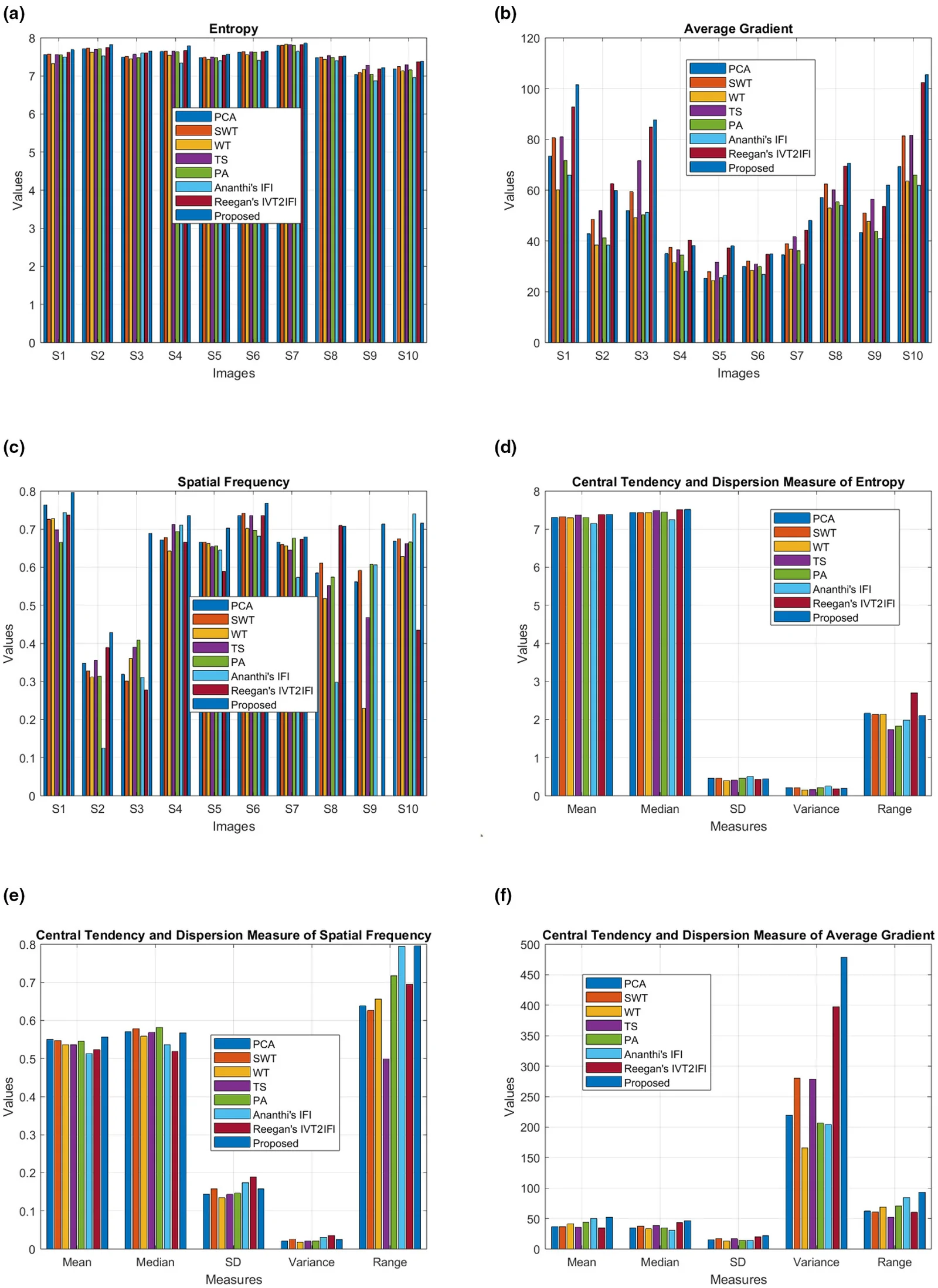
Graphical analysis of performance and statistical analysis with SOTA. **(a)** Performance analysis via entropy (Table 1). **(b)** Performance analysis via AG (Table 2). **(c)** Performance analysis via SF (Table 3). **(d)** Statistical analysis of entropy (Table 4). **(e)** Statistical analysis of SF (Table 5). **(f)** Statistical analysis of AG (Table 6).

**Figure 15:**
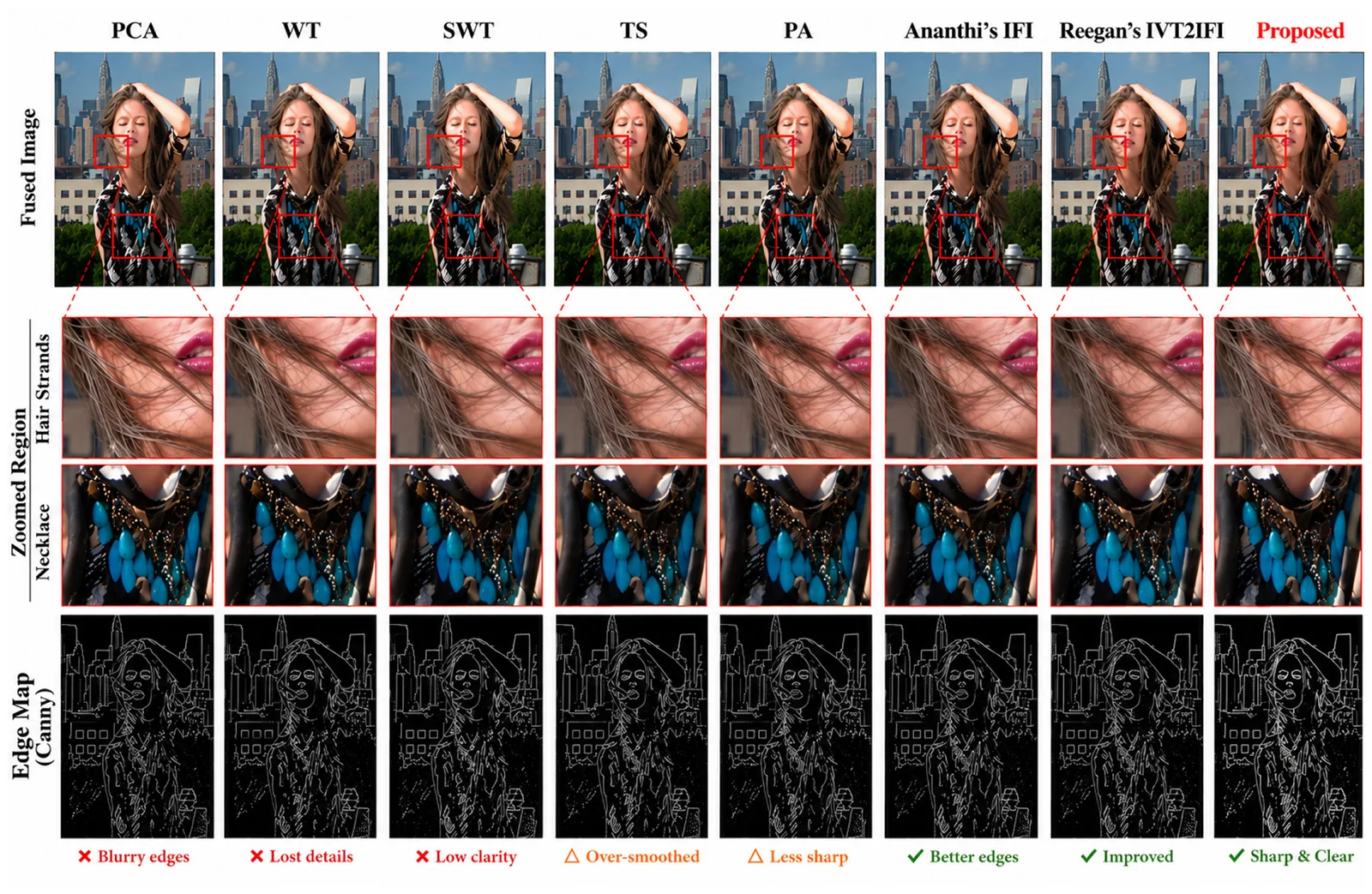
Visual comparison of the image S4 along existing fusion methodologies with zoomed-in details and edge map analysis.

Finally, Table 3 present the SF values via Figure 14c, in which SF indicates the similarity between images fused with the rate of change in intensity from one pixel to another. By Section 2.4.4, a high SF-valued image is considered less noisy and a standard texture image. Most of the images, S1–S7, S9 achieves the highest SF value and S8, S10 secures the second highest AG value. To validate the impact of the SF on images, SF is statistically evaluated with all other metrics via central tendency and dispersion measure which is displayed in the Table 6 and Figure 14c.

In this comparative analysis, E, AG, and SF for the results of the proposed methodology with seven other conventional and recent methods (Ananthi and Balasubramaniam, 2015; Bavirisetti and Dhuli, 2016; Chai et al., 2017; Diwakar et al., 2021; Jebadass and Balasubramaniam, 2023; Kaur et al., 2021; Li et al., 2018) evaluated using the central tendency measure (mean & median) and dispersion measure (SD, variance & range) and represented in the Tables 4–6. It shows that across all three metrics, E, AG, and SF, which are evaluated through the statistical analysis, the proposed method consistently outperforms existing fusion techniques by excelling in the mean and median. It occasionally exhibits controlled variability via SD, variance, and range. Hence, this methodology can be adapted for enhanced dynamic performance by proving both effectiveness and efficiency by striking a strong balance between high detail preservation and consistency across fused images.

The proposed fusion technique achieves optimal level values for all the performance metrics, which is deliberately explained graphically through Figure 14. The resultant image signifies better visual perception by utilizing this kind of versatile fusion strategy.

The bold values in the metrics table indicate the effectiveness of the proposed fusion technique, and red, green, blue colors indicate the first, second, and third highest metric values. Considering the experimental results and quality measures, it is clear that this technique consistently outperforms other existing methods, delivering enhanced contrast, brightness, and sharpness levels.

## 5 Conclusion

In contrast to existing state-of-the-art methods, the proposed intuitionistic fuzzy-based OBPDS algorithm turns into an organized image fusion strategy. In this study, first, the input images are converted from crisp into FI's, then the FI's are converted into IFI's. After that, the images are divided into blocks by optimized block-based partition, and they are converted into blurred images as *G*IFI's by image defocusing. Finally, the fused output images {*S*1, *S*2, *S*3, ⋯ } are formed by the blocks-image synthesis process followed by defuzzification. Further, the output images are compared with various fusion algorithms such as (Ananthi and Balasubramaniam, 2015; Bavirisetti and Dhuli, 2016; Chai et al., 2017; Diwakar et al., 2021; Jebadass and Balasubramaniam, 2023; Kaur et al., 2021; Li et al., 2018). The performance is evaluated under entropy, AG, and SF of the images along with their statistical analysis using centrality and dispersion measures, which affirms a better result for the proposed method. In future work, this study can be employed by utilizing further extensions of intuitionistic fuzzy sets, facilitating the fusion of more intricate images.

## Data Availability

Publicly available datasets were analyzed in this study. This data can be found here: https://github.com/xingchenzhang/MFIF.
